# Fabrication of 1 × *N* integrated power splitters with arbitrary power ratio for single and multimode photonics

**DOI:** 10.1515/nanoph-2023-0694

**Published:** 2024-01-24

**Authors:** Jack Haines, Valerio Vitali, Kyle Bottrill, Pooja Uday Naik, Marco Gandolfi, Costantino De Angelis, Yohann Franz, Cosimo Lacava, Periklis Petropoulos, Massimiliano Guasoni

**Affiliations:** Optoelectronics Research Centre, University of Southampton, Southampton, England; Department of Information Engineering, University of Brescia, Brescia, Italy; Istituto Nazionale di Ottica – Consiglio Nazionale delle Ricerche, Via Branze 45, Brescia, 25123, Italy; Consorzio Nazionale Interuniversitario per le Telecomunicazioni (CNIT), Viale G.P. Usberti 181/A, 43124 Parma, Italy; Department of Electrical, Computer and Biomedical Engineering, University of Pavia, Pavia, Italy

**Keywords:** integrated photonics, power splitting, multimode, silicon nitride

## Abstract

Compact power splitters are essential components in integrated optics. While 1 × 2 power splitters with uniform splitting are widely used, a 1 × *N* splitter with arbitrary number *N* of ports and arbitrary splitting ratio is yet to be demonstrated. In this work we address this problem. We fabricate and characterise 1 × *N* integrated power splitters that provide fully arbitrary splitting ratios. The core of our design is represented by an array of *N* non-equally spaced waveguides fabricated on a silicon nitride-on-insulator wafer. Any arbitrary 1 × *N* splitting ratio can be achieved by properly setting the array length and the dimension of the (*N*–1) nano-gaps between the adjacent waveguides. Most importantly, at variance with state-of-the-art solutions, our devices can be designed for arbitrary splitting of higher-order modes. In this manuscript we provide the first experimental demonstration of 1 × *N* arbitrary splitting ratio for both the fundamental modes (TE00 and TM00) and the TE01 mode, here up to *N* = 5 ports. With a footprint of 20 μm^2^/port, a bandwidth up to 70 nm and an excess losses <0.2 dB, our devices set a new benchmark for optical power splitters in both standard single-mode photonics as well as in the emerging integrated multimode photonics technology, and may therefore boost key photonic applications, from optimal power distribution and equalization up to signal processing operations.

## 1 Introduction

Integrated optical power splitters are essential components in photonics. While the design of 1 × 2 splitters providing uniform splitting at the 2 output ports is relatively straightforward, the implementation of 1 × *N* splitters with arbitrary number of *N* output ports and with arbitrary splitting ratio discloses significant challenges in terms of compactness, complexity, scalability and excess losses (ELs). Indeed, a general solution to this problem is yet to be demonstrated. On the other hand, these splitters may enable or boost key applications that benefit from multiple output ports and/or non-uniform splitting, including optical feedback and signal monitoring [1], optimal power distribution [2], [3], equalization [4], and network access [5], to name a few. In addition, they may provide a new route to complex all-optical logical and signal processing operations on chip [6], [7], [8], [9].

Typical performance indicators for power splitters include footprint, design complexity, arbitrary splitting, scalability, EL and bandwidth. With the advent of multimode photonics [10], [11], [12] that has characterized the last decade, a further key feature of new-generation power splitters will be the ability to split the power of higher-order modes. First of all, this would allow replicating the above-mentioned applications by using higher-order modes, thus increasing the overall throughput and efficiency. Furthermore, it may enable the implementation of new operations for multimode optical networks, such as selective optical switching based on the modal content.

State-of-the-art technology in photonic integrated circuits is mainly based on directional couplers, Y-junctions and multimode interferometer (MMI) devices, which have been widely used to implement 1 × 2 splitters. Typically, on chip 1 × *N* splitters with *N* > 2 output ports of equal splitting ratios (equal power at all the output ports) are realised via cascaded devices, namely, Y-junctions [13], [14], [15] or MMIs [16], [17]. However, these cascaded schemes come at the expense of large EL (directly proportional to the number of cascaded stages at best), bandwidth reduction (inversely proportional to the number of stages) and a significantly large footprint. Moreover, any fabrication error in one splitter will affect its whole branch, which poses a limit to the splitting uniformity. Standard 1 × *N* MMIs have a smaller footprint than cascaded Y-junctions but their design is substantially more complex and hardly scalable [17] even in the simplest case of equal splitting ratios.

The design of ultra-compact splitters is considerably challenging, especially if arbitrary splitting is targeted. A few integrated MMIs have recently been proposed to achieve arbitrary 1 × 3 [18] and uniform 1 × 4 [19] power splitting in an extremely compact design. These are based on complex and unconventional geometries where light undergoes scattering and reflection in several locations whose design requires numerical optimization of tens to hundreds of parameters for a total computational time that can exceed 100 h. This may compromise the beam quality and mode purity at the output as well as the ability to scale the design to 1 × *N* schemes with arbitrary power ratio and number of output ports.

In this work we fabricate and characterize a new integrated platform for 1 × *N* power splitters, which is based on the theoretical guidelines and design procedures that we have previously reported in Ref. [20]. Given an array of *N* coupled and non-equally spaced waveguides, any arbitrary 1 × *N* splitting ratio can be achieved by properly setting the array length and the dimension of the (*N*–1) nano-gaps between the *N* adjacent waveguides.

The keystone of our platform is its simple design that reduces drastically the computation time and results in a straightforward fabrication process that provides consistently the expected splitting ratios in all the cases under test. Most importantly, the complexity of the design is independent of the specific splitting ratio or number of ports, which makes our platform fully flexible and easily scalable. The strong coupling among adjacent waveguides and the absence of discontinuities lead to effective splitting in a compact footprint, with low EL and high beam quality. Moreover, differently from standard power splitters, our platform can be used to split higher-order modes besides the fundamental one, again at no-extra cost in terms of complexity. Note that in principle higher-order mode splitting could be obtained by cascading a mode converter (from higher-order mode to fundamental mode), a splitter (for fundamental mode) and then a further mode-converter (from the fundamental mode to the initial higher-order mode). However, this cascade scheme would substantially increase the total footprint and complexity and conversely would reduce the bandwidth and output modal purity.

Some recent works have experimentally investigated the design of 1 × 2 splitters for higher-order modes with equal splitting ratios [21], [22], [23], [24], whereas the theoretical analysis of 1 × 2 splitters with arbitrary splitting ratio has been originally introduced in Ref. [25]. In this manuscript, we provide the first experimental demonstration of 1 × *N* splitting with an arbitrary splitting ratio for both the fundamental (TE00, TM00) modes as well higher-order modes (here TE01).

In Section 2 we provide an overview of the design and fabrication; in Sections 3 and 4 we report on a variety of 1 × 3 and 1 × 5 arrays with arbitrary splitting ratios for the TE00, TM00 and TE01 modes; in Section 5 we compare our platform against state-of-the-art approaches and we discuss how our current design could be modified to enhance the overall performance and to integrate novel applications.

## 2 Design and fabrication

**Figure 1:**
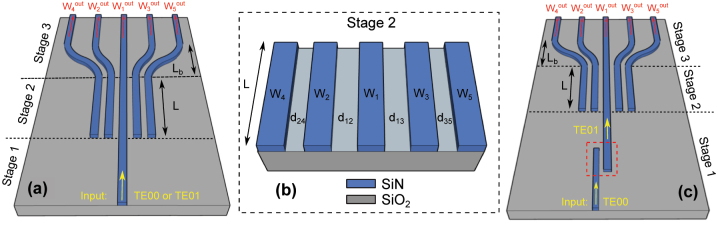
Schematic of the power splitters and related stages discussed in this work. Here, the example of 1 × 5 splitters with TE00 or TE01 excitation is illustrated. Two different configurations are implemented. In panel (a) the input beam – coupled either to the TE00 or TE01 mode – is launched in the central waveguide *W*_1_ at the input of stage-1, and is split in the following stages. The device works therefore as a pure power splitter for TE00 or TE01 beams. In panel (c) the input beam – coupled to the mode TE00 – is first converted to TE01 via the directional coupler (highlighted by the red dashed rectangle) in stage-1 and it is then split in the following stages. Mode conversion and splitting are therefore integrated in the same device. Panel (b) is a magnification of stage-2 reporting the notation used in this work. While in general the gaps can all be different from each other, in this work *d*_12_ = *d*_13_ and *d*_24_ = *d*_35_. Note that the outer waveguides *W*_4_ and *W*_5_ are not present in 1 × 3 splitters.

In order to show arbitrary power splitting of both the fundamental and higher-order modes, we have designed, fabricated and tested several power splitters for TE00, TM00 and TE01 modes. In this work the waveguides core is made of stoichiometric silicon nitride (SiN) and is buried in silica (SiO_2_) cladding. While in principle we may design splitters for any number of output ports and any higher-order mode, here we focus on devices with *N* = 3 or *N* = 5 output ports and we choose TE01 as the higher-order mode to be split. Indeed, this simplifies the free-space input coupling and the output modal decomposition. In the following, we indicate with 1 × *N*-TE00, 1 × *N*-TM00 and 1 × *N*-TE01 (*N* = 3, 5) the splitters for TE00, TM00 and TE01 modes, respectively. Figure 1 shows a schematic of the device, which is composed of 3 distinct stages. In order to use the same notation for 1 × 3 and 1 × 5 splitters, here *W*_1_ indicates the central waveguide, *W*_2_ and *W*_3_ represent the pair of inner waveguides and *W*_4_ and *W*_5_ represent the pair of outer waveguides. Similarly, we use the notation $W_{n}^{\text{out}}$ (*n* = 1, 2, 3, 4, 5) for the output port corresponding to the output end of waveguide *W*_*n*_.

Stage-1 is the coupling stage where the input beam to be split is launched in the central waveguide *W*_1_. Two different scenarios are explored to couple TE01 beams, both of which correspond to distinct practical applications. In the first case, we implement free-space mode conversion via an external phase-plate (PP). The incident light at the input of stage-1 is therefore a TE01-like beam and our device works purely as a power splitter (see Figure 1a). Alternatively, mode conversion can be realised through an on chip asymmetric directional coupler in stage-1 (see Figure 1c). In this latter case, the device represents a mode converter and splitter fully integrated on the same platform.

Stage-2 is the core of our power splitter. It is composed of an array of *N* coupled waveguides that split the beam in input from the central waveguide *W*_1_ into *N* outputs [20]. Each waveguide of the array is coupled to the nearest neighbour waveguides. The overall power exchange between the waveguides is defined by the array length and by the coupling coefficients between neighbouring waveguides. Therefore, given a target output splitting ratio at a fixed reference wavelength, the inverse design consists of calculating the corresponding array lengths and the coupling coefficients. Finally, since each coupling coefficient between neighbouring waveguides is related to the width of the gap between them, we can simply calculate the latter. In general, the resulting gap widths are different from each other, namely, the waveguides are not equally spaced. Here we use the notations *d*_12_ and *d*_13_ to refer to the widths of the inner gaps, whilst *d*_24_ and *d*_35_ represent the widths of the outer gaps. Note that the design requires the knowledge of the materials refractive index at the reference wavelength, both of which are reported in the Supplementary Material Section 1.

Stage-3 is not essential but is implemented to separate the output ports by at least 10 μm, which facilitates both the power measurements and the modal decomposition at each output port.

Table 1 reports the characteristics of the power splitters discussed in this work along with the corresponding geometrical parameters. The splitting ratios and the corresponding reference wavelengths are not the same in the different splitters under investigation as we want to highlight our ability to set completely arbitrary values. All the splitters under test are symmetric with respect to the central waveguide, that is, *d*_12_ = *d*_13_ and *d*_24_ = *d*_35_. This allows for the assessment of the fabrication and measurement precision.

**Table 1:** List of power splitters presented in this work.

Splitter	Figure	Power ratio	RW (nm)	*d*_12_ (nm)	*d*_24_ (nm)	*L* (μm)	*R*_b,in_ (μm)	*R*_b,out_ (μm)	*L*_b_ (μm)
1 × 5-TE00	4a	4.5:1:9:1:4.5	1570	300	449	23.59	60	40	52.05
1 × 5-TE00	4b	1:1:2:1:1	1560	300	449	19.99	60	40	52.05
1 × 3-TE01	6a	1:1:1	1535	300	–	10.84	40	–	36.30
1 × 3-TE01	6b	3.5:1:3.5	1570	300	–	15.64	40	–	36.30
1 × 5-TE01	7a	2.5:1:6:1:2.5	1575	300	464	28.95	60	40	49.56
1 × 5-TE01	7b	1:2:2:2:1	1565	300	464	24.12	60	40	49.56

RW = reference wavelength. Parameters related to stage-2 are: *d*_12_ = inner gap; *d*_24_ = outer gap; L = array length. Note that in this work *d*_13_ = *d*_12_ and *d*_35_ = *d*_24_. Parameters relate to stage-3 are: *R*_b,in_ = bend radius of inner waveguides *W*_2_ and *W*_3_; *R*_b,out_ = bend radius of outer waveguides *W*_4_ and *W*_5_; *L*_b_ = length of the bent section. The power ratio indicates the relative power at the output ports of the array. For example, a 1 × 5 arrays with the ratio – 4.5:1:9:1:4.5 – indicates that the outer ports ( $W_{4}^{\text{out}}$ and $W_{5}^{\text{out}}$ ) have 4.5 times the power of the inner ports ( $W_{2}^{\text{out}}$ and $W_{3}^{\text{out}}$ ) and the central port ( $W_{1}^{\text{out}}$ ) has 9 times the power of the inner ports.

Indeed thanks to the symmetric geometry we should find equal power splitting at the pair of inner ports ( $W_{2}^{\text{out}}$ and $W_{3}^{\text{out}}$ ) and at the pair of outer ports ( $W_{4}^{\text{out}}$ and $W_{5}^{\text{out}}$ ), respectively. The waveguides are 300 nm in height with a width of *w* = 1 μm for 1 × *N*-TE00 and 1 × *N*-TM00 splitters in order to fulfil the single-mode condition (only TE00 and TM00 supported) in the range of wavelengths of interest, namely 1530–1610 nm band. The width is increased to *w* = 2 μm for the 1 × *N*-TE01 splitters as TE01 is also supported at this width.

**Figure 2:**
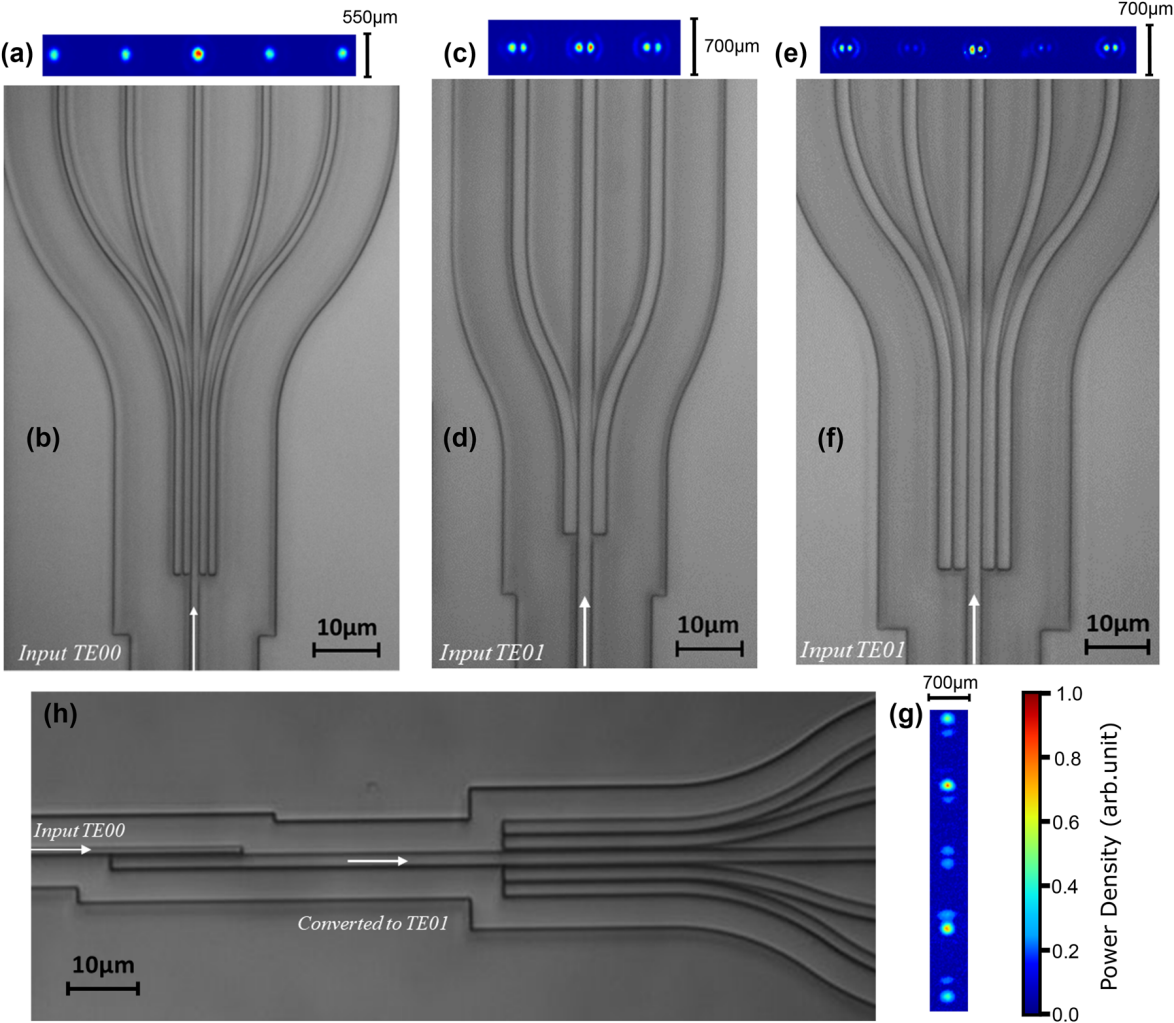
Microscope images of several different devices fabricated and tested (b, d, f, h), along with camera images of the corresponding near-field at the output ports (a, c, e, g). Panels (a, b) display the 1 × 5-TE00 power splitter for 1:1:2:1:1 splitting, see results in Figure 4b. Panels (c, d) display the 1 × 3-TE01 power splitter for equal splitting ratios, see results in Figure 6a. Panels (e, f) display the 1 × 5-TE01 power splitter for 2.5:1:6:1:2.5 splitting, see results in Figure 7a. Panels (g, h) display the 1 × 5-TE01 power splitter with integrated mode converter whose preliminary results are discussed in the Supplementary Material, Sections 2 and 3.

The fabrication has been developed through the CORNERSTONE prototyping service based at the University of Southampton. The devices under test were fabricated on a SiN-on-insulator wafer starting from a 300 nm thick layer of SiN deposited onto an 8-inch Si wafer with a 3 μm thick SiO_2_ layer. As the minimum feature size is ∼250 nm, the geometry is patterned via deep-ultraviolet (DUV) lithography rather than e-beam lithography. Patterning is then followed by an inductively coupled plasma etch (ICP) process and a 2 μm thick SiO_2_ capping layer deposition. End facet polishing is finally implemented to maximise input and output coupling efficiency. The devices are imaged by a 100× microscope and are shown in Figure 2 along with the corresponding near-fields at the output ports. An enlarged version of the near-field images is provided in the Supplementary Material, Section 3.

## 3 Experimental results on 1 × *N*-TE00 and 1 × *N*-TM00 splitters

**Figure 3:**
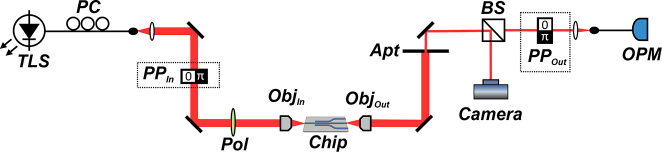
Experimental setup. TLS = tunable laser source (1530–1610 nm); PC = polarisation controller; pol = linear polariser; Obj_In,Out_ = input/output objective; Apt = aperture; BS = beam splitter; OPM = optical power meter. The input phase plate PP_In_ is used for free-space excitation of the TE01 mode in 1 × 3-TE01 and 1 × 5-TE01 splitters, otherwise is not present. Similarly, the output phase-plate PP_Out_ is used for modal decomposition at the output.

A schematic of the experimental setup for the characterisation of the fabricated devices is shown in Figure 3. A narrow band CW tunable diode laser with 10 dBm output power across the 1530–1610 nm spectral region is used as the optical source. The light is coupled into the central waveguide *W*_1_ via edge-coupling through a 60×, 0.85NA objective (Obj_In_). A polarisation controller (PC) combined with a linear polariser (Pol) precisely control the excitation of either the TE or TM waveguide modes with a polarisation extinction ratio exceeding 20 dB. The chip including the power splitters under test is positioned on an *XYZ* piezoelectric stage for high-precision optical coupling. At the output, light is collected through a second 60×, 0.85NA objective (Obj_Out_). The beam from each output port is isolated using an aperture (Apt) and the corresponding power measured with an optical power meter with 1 nW resolution. The near-field at each output port is imaged on an infrared camera.

**Figure 4:**
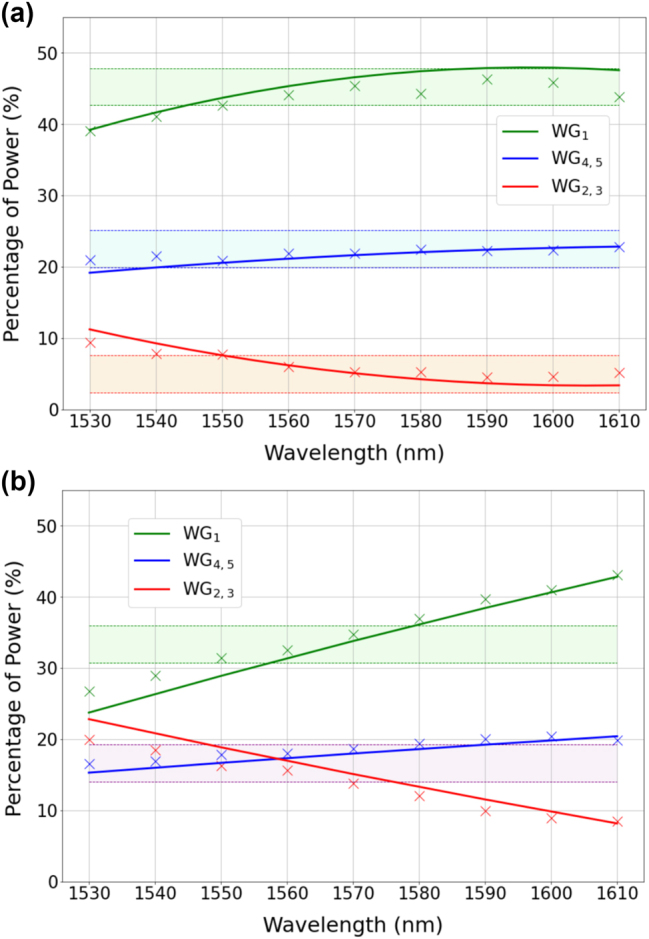
Measured (crosses) and simulated (solid lines) splitting ratios at the output ports of the 1 × 5-TE00 power splitters reported in Table 1. The shaded regions indicate a ±2.5 % variation with respect to the nominal splitting ratios, namely 4.5:1:9:1:4.5 for panel (a) (in percentage: 22.5 %, 5 %, 45 %, 5 %, 22.5 %) and 1:1:2:1:1 for panel (b) (in percentage: 16.66 %, 16.66 %, 33.33 %, 16.66 %, 16.66 %). To simplify the representation, the experimental data related to ports *W*_3_ (*W*_5_) have been omitted in the figures, as they closely resemble the data at ports *W*_2_ (*W*_4_), with differences as small as 1 %. Therefore, the notation *W*_2,3_ (*W*_4,5_) is employed in the legend to indicate that the reported data applies to both waveguides *W*_2_ and *W*_3_ (*W*_4_ and *W*_5_).

The first splitter under test is a 1 × 5-TE00 that is designed to provide a splitting ratio of 4.5:1:9:1:4.5 at the reference wavelength of 1570 nm (see Table 1). The experimentally measured splitting ratios are reported in Figure 4a along with the results obtained by finite-element-method (FEM) numerical simulations of the device via COMSOL Multiphysics. The bandwidth is calculated with respect to a ±0.5 dB variation of an equal power splitting, that is, when the power is the same at the output ports. This enables a fair comparison between the bandwidth of splitters having different numbers of output ports and different splitting ratios. In the case of 1 × 5 splitters, this corresponds to a ±2.5 % variation. The shadowed regions in Figure 4a indicate the upper and lower limits of the ±2.5 % variation, from which we estimate a bandwidth of ∼60 nm (1550–1610 nm band). It should be noted that the bandwidth is essentially related to chromatic dispersion. The latter is responsible for the wavelength dependence of the coupling coefficients, which ultimately define the dynamics of the power exchange between the waveguides of the array. Therefore, the lower the chromatic dispersion, the broader the bandwidth.

The second 1 × 5-TE00 splitter is designed to provide a splitting ratio of 1:1:2:1:1 at the reference wavelength of 1560 nm. Experimental results and numerical simulations are reported in Figure 4b. Similarly to the previous case shown in Figure 4a, we note a significant agreement between simulations and experiments. However, the bandwidth is reduced to ∼20 nm (1550–1570 nm band). In the Supplementary Material, Section 2, we report on a 1 × 5-TM00 splitter we have fabricated that exhibits equal splitting ratio at the output ports over the tested spectral window (1530–1610 nm).

In general, our design of 1 × 5-TE00 and 1 × 5-TM00 splitters turns out to be robust and reliable for any arbitrary power ratio. Note that all the output beams exhibit a clean and circular shape typical of the fundamental mode (see e.g. Figure 2a). It is also worth noting a good quantitative agreement between experiments and simulations across the whole 1530–1610 nm spectral region, which attests to the predictability of our theoretical model.

The reason for the good agreement is twofold. First, the deposition of SiN in our cleanrooms is a well-established fabrication process and we could rely on the precise knowledge of the SiN refractive index for our design. Second, the use of DUV lithography allows the definition of nano-gaps with a typical maximum deviation of 20 nm from the nominal values. These are key factors since the core of our design relies on the estimation of the coupling coefficients between adjacent waveguides, which depend critically on both the refractive index and the nano-gap widths. Indeed, according to the numerical simulations, variations of ±20 nm in the gaps width do not affect significantly the splitting ratios and the operational bandwidth of our power splitters, which proves the robustness of our approach to fabrication variations.

As anticipated in the previous section, further evidence of the fabrication accuracy is provided by the symmetric power splitting ratios at the inner and outer output ports. The difference between ports $W_{2}^{\text{out}}$ and $W_{3}^{\text{out}}$ is typically smaller than 1 % (see Table S1 in Supplementary Material Section 4) and the same applies to the outer ports $W_{4}^{\text{out}}$ and $W_{5}^{\text{out}}$ .

Because it may be challenging to measure a low EL, the assessment is carried out with two distinct techniques in order to provide an accurate estimate. In the first case, the EL is calculated as the ratio between the average transmission of the splitter and the average transmission of a reference straight waveguide with the same dimensions of the waveguides in the splitter [26]. The above-mentioned average transmissions are performed over 5 different replicas of the splitter under test and of the reference waveguide.

**Figure 5:**
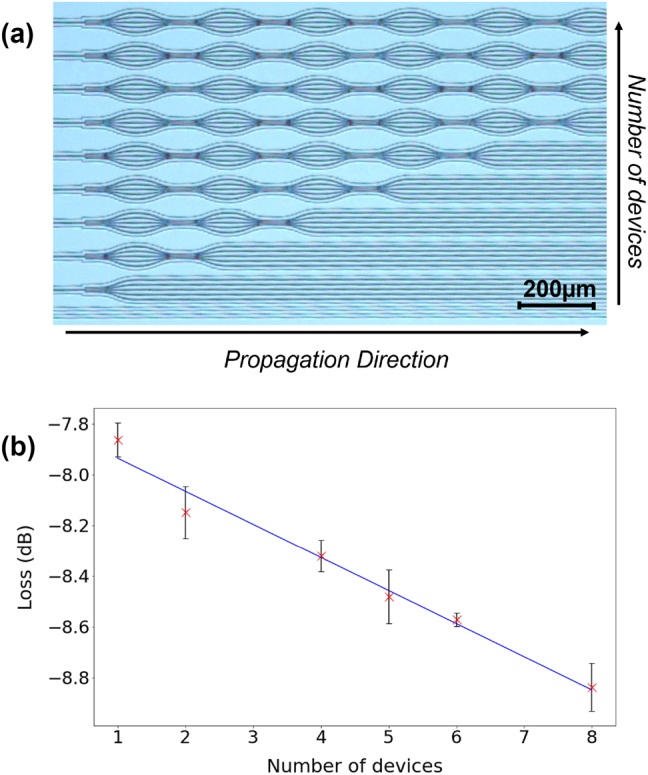
Estimation of excess loss via in-series cascade of splitters at a wavelength of 1550 nm. Panel (a) displays a microscope image of the in-series-cascades. Different rows include a different number of cascaded splitters. By measuring the transmitted power at each row, the total loss is computed versus number of cascaded devices, which is reported in panel (b) (red crosses). The slope of the fitted line (solid blue) indicates the excess loss of a single splitter, here ∼0.13 dB/device. Measurements related to rows 3 and 7 are omitted due to visible damage at their end facets.

In the second case, the EL is calculated via a system of in-series-cascaded splitters [27], [28], as illustrated in Figure 5. The transmission is evaluated as a function of the number of cascaded splitters and then fitted with a straight line, whose slope indicates the EL. In both cases, we estimate an EL as small as <0.2 dB, which comes as the result of a smooth geometry and the absence of discontinuities at the interface between the different stages.

## 4 Experimental results on 1 × *N*-TE01 splitters

One of the advantages of our approach based on arrays of waveguides is that splitting higher-order modes does not bring any additional complexity to the design and fabrication process [20]. On the other hand, two additional steps are introduced in the experimental setup, namely, the selective excitation of higher-order modes at the input (stage-1) as well as the mode decomposition to estimate the modal extinction ratio (MER) at the output ports. As anticipated, in this work the chosen higher-order mode is TE01, which is launched via free-space optics or through integrated conversion on chip. In the first case we employ a standard fused silica PP with 0–*π* phase-step (PP_in_ in Figure 3), which is commonly used to excite TE01 modes with high modal purity [29].

Mode decomposition follows a standard procedure detailed in Refs. [29], [30], this involves passing a beam through an additional PP (PP_out_ in Figure 3) and coupling the transmitted light into a single-mode fibre. By decomposing the beam at the output port $W_{n}^{\text{out}}$ we estimate the fraction of beam power *P*_00_ and *P*_01_ carried by the mode TE00 and TE01, respectively. We indicate with MER_*n*_ the MER evaluated at the output port $W_{n}^{\text{out}}$ , which is the ratio *P*_01_/*P*_00_ between the above-mentioned powers.

The measure of the MER at each output port is one of the key performance indicators in our 1 × *N*-TE01 splitters. A large MER indicates high modal purity, namely, that most of the power is coupled to the TE01 mode. Conversely, a low MER would indicate that our 1 × *N*-TE01 splitter is affected by a substantial residual TE00 component. This may be either the consequence of inefficient selective excitation of the TE01 mode at the input coupling end or of modal conversion from TE01 to TE00 inside the splitter due to bending or geometry imperfections. As in the case of the excess loss estimation, we use a reference straight waveguide to evaluate the excess MER degradation induced by the splitter itself, beyond the inherent MER degradation due to coupling. The MER of the reference waveguide is MER_ref_ ∼ 14 dB.

The first 1 × 3-TE01 splitter under test is designed to provide equal splitting ratios at 1535 nm. Here, the average MER measured at the output ports is ∼11 dB, which corresponds to an excess MER degradation of 3 dB. According to our simulations, this is due to a partial re-conversion TE01-to-TE00 in the curved sections of stage-3 and may be reduced by increasing the bending radii. Note that, as previously mentioned, stage-3 is redundant and introduced in our setup only to simplify the measurements.

**Figure 6:**
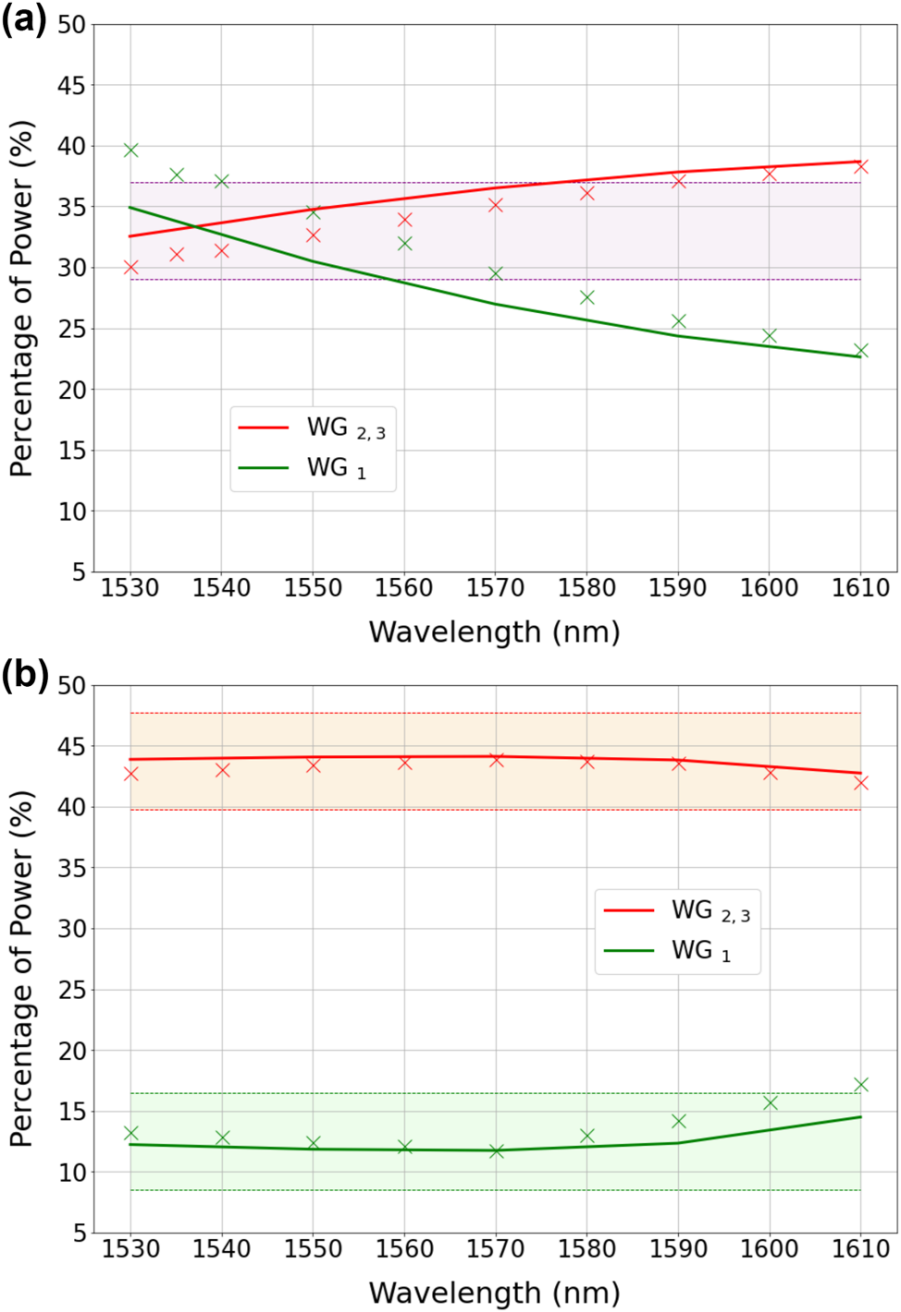
Measured (crosses) and simulated (solid lines) splitting ratios at the output ports of the 1 × 3-TE01 power splitters reported in Table 1. The shaded regions indicate a ±4 % variation with respect to the nominal splitting ratios, namely 1:1:1 for panel (a) (equal splitting) and 3.5:1:3.5 for panel (b) (in percentage: 43.75 %, 12.50 %, 43.75 %). To simplify the representation, the experimental data related to ports *W*_3_ have been omitted in the figures, as they closely resemble the data at ports *W*_2_, with differences as small as <1% . Therefore, the notation *W*_2,3_ is employed in the legend to indicate that the reported data applies to both waveguides *W*_2_ and *W*_3_.

The results are illustrated in Figure 6a and show a good quantitative agreement between experiments and FEM simulations. As in the examples discussed in the previous section, the bandwidth is calculated with respect to a ±0.5 dB variation of an equal power splitting. In the case of 1 × 3 splitters, this corresponds to a ±4 % variation. In this example, the measured bandwidth is ∼30 nm (1540–1570 nm band). The output beams imaged on the camera are displayed in Figure 2c, where we distinctly observe the characteristic shape of the TE01 mode at each output port.

Good agreement is found also in the second 1 × 3-TE01 splitter illustrated in Figure 6b and designed to provide 3.5:1:3.5 splitting ratio at a reference wavelength of 1570 nm, for which the bandwidth is at least 70 nm (1530–1600 nm band) and the MER degradation is similar to the previous case.

**Figure 7:**
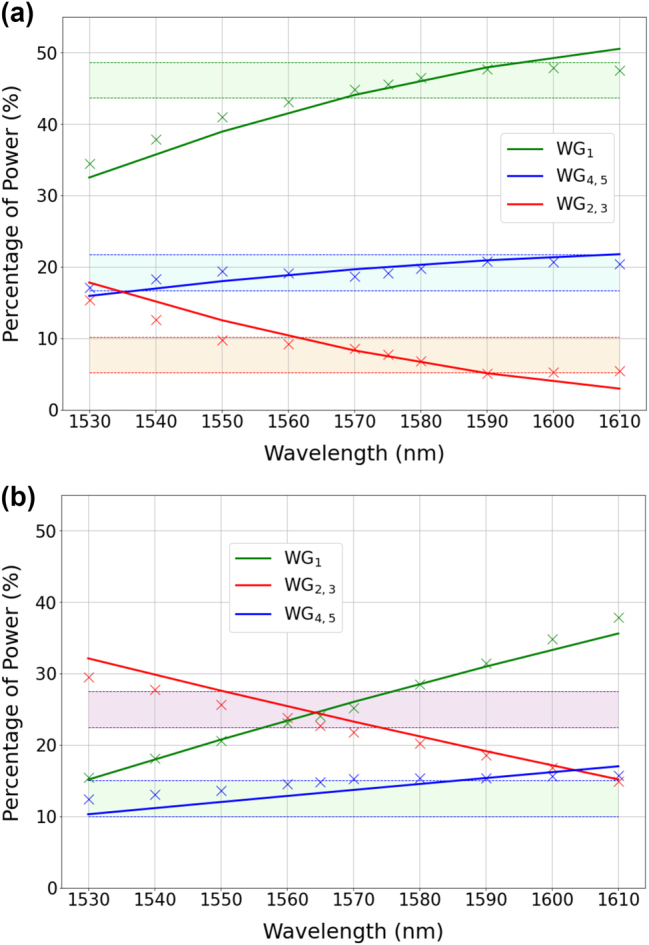
Measured (crosses) and simulated (solid lines) splitting ratios at the output ports of the 1 × 5-TE01 power splitters reported in Table 1. The shaded regions indicate a ±2.5 % variation with respect to the nominal splitting ratios, namely 2.5:1:6:1:2.5 for panel (a) (in percentage: 19.23 %, 7.69 %, 46.15 %, 7.69 %, 19.23 %) and 1:2:2:2:1 for panel (b) (in percentage: 12.5 %, 25 %, 25 %, 25 %, 12.5 %). To simplify the representation, the experimental data related to ports *W*_3_ (*W*_5_) have been omitted in the figures, as they closely resemble the data at ports *W*_2_ (*W*_4_), with differences as small as <1% . Therefore, the notation *W*_2,3_ (*W*_4,5_) is employed in the legend to indicate that the reported data applies to both waveguides *W*_2_ and *W*_3_ (*W*_4_ and *W*_5_).

Similar considerations and results are found for the 1 × 5-TE01 splitters reported in Figure 7, providing 2.5:1:6:1:2.5 and 1:2:2:2:1 splitting at the reference wavelengths of 1575 nm and 1565 nm respectively. In the Supplementary Material Sections 2 and 3, we report some preliminary result on the 1 × 5-TE01 splitter with an integrated mode converter displayed in Figure 2g and h.

## 5 Conclusion and perspectives

We have designed, fabricated and characterized a novel platform for integrated optical power splitting that is based on an array of coupled and non-equally spaced waveguides. Our approach merges compactness along with a simple and reliable design strategy that allows the use of DUV lithography rather than e-beam lithography, resulting in a more cost-effective and faster fabrication process. The core of our device is indeed represented by stage-2 which requires the optimization of just *N* parameters (length and *N*–1 nano-gaps) to achieve fully arbitrary 1 × *N* power splitting. The optimization algorithm is based on the solution of a system of *N* coupled linear differential equations and requires therefore a remarkably low computational effort [20]. The flexibility of our platform manifests itself not only in the ability to provide arbitrary power splitting for an arbitrary mode, but also in the possibility to reshape the design to extend the range of applications. For example, our splitters could be trivially modified to accommodate multiple input modes and ports. A variety of different arbitrary splitting ratios could be therefore obtained by selectively exciting different input modes and/or ports in stage-1, as reported in the Supplementary Material Section 4. A further remarkable perspective is the implementation of all-optical logical and mathematical operations on chip, which may be pursued by properly exciting simultaneously several input ports.

Moving forward, limitations on the number of modes and output ports will be explored. While the complexity of the design is independent of the higher order mode to be split and almost independent of the number of output ports, it is however still unclear at what extent the design scalability translates into manufacturing scalability. Certainly, it is not difficult to fabricate arrays with tens of waveguides, with individual waveguides large enough to support the desired higher-order mode. However, we are still investigating what this entails in terms of fabrication robustness.

In Table 2 we compare the main key-performance indicators in our platform and in some relevant and ultra-compact power splitters recently reported and experimentally characterized. Note that while 1 × *N* splitters (*N* > 2) have been implemented via a cascade of Y-junctions and standard MMIs [13], [16], they do not offer higher-order mode operation nor arbitrary splitting, and in addition they are characterised by large EL and a footprint that is several orders of magnitude larger than the devices reported in Table 2.

**Table 2:** Comparison between recent state-of-the art works and our platform.

Ref	Type	BQ	FP (μm^2^/port)	Complexity (par/port)	Split	EL (dB)	BW (nm)	Multimode
[31]	Y-junction	High	1.6	4	1 × 2 Arb	0.5	40	No
[18]	MMI	Low	4.3–6.5	300–450	1 × 2, 1 × 3 Arb	1	<30	No
[19]	MMI	Low	10.8	>40	1 × 4 equal	<1^a^	15	No
[21]	Dir. coupler	High	15^b^	1^b^	1 × 2 equal	0.2–2.1	<30	Yes
[22]	Y-junction	High	180	7	1 × 2 equal	0.5	80	Yes
[23]	MMI	NA^c^	5000	2	1 × 2 equal	1.24–1.85	60	Yes
THIS	AWG	High	20^d^	1	1 × 3, 1 × 5 Arb	<0.2	15–70	Yes

All the data in the table are either directly reported or otherwise inferred. BQ = beam quality; FP = footprint; EL = excess loss; BW = bandwidth; Arb = arbitrary splitting ratios; equal = equal splitting ratios only (non arbitrary). Notes: ^a^Excess loss not reported but insertion loss of 1.08 dB mentioned; ^b^the core of the device is the coupling region ∼15 μm × 2 μm, whose parameters are length and gap; ^c^beam quality not clear from the data reported; modal extinction ratio not provided; ^d^refer to the footprint of stage-2, where splitting takes actually place. Stage-3 is indeed not essential and introduced in this work only to simplify the experimental measurements.

The complexity is evaluated in terms of the number of parameters to optimize. Both the complexity and the footprint are normalised with respect to the number of output ports. The beam quality refers to the beam shape at the output ports. It is worth noting that in Refs. [18], [19], the power splitting is assisted by multiple reflections and superposition of many scattered waves that is likely to result in speckled, spatially incoherent output beam shapes.

To the best of our knowledge, so far 1 × 3 splitters with arbitrary power ratios have been reported for single-mode operation only, and 1 × 2 splitters with equal splitting ratios for multimode operation. Along with the unique feature of providing arbitrary splitting with *N* > 3 ports, our platform represents the first demonstration of arbitrary splitting of higher-order modes. It should be noted that the DUV lithography process in our cleanrooms sets a limit of ∼250 nm to the minimum gap-width. The latter could be reduced via e-beam lithography, which would result in a stronger coupling among waveguides and lead to an even shorter coupling length. This would reduce the footprint by a factor of 2 at least.

Finally, unlike some recently reported works that rely on intense computation to optimise a considerable number of parameters (120 h to optimize 900 parameters in Ref. [18]), in our splitters the complexity is drastically reduced (1 parameter/port) and independent of the spatial mode to be split, of the number of output ports and the splitting ratio. This results in effortless design, scalability and good tolerance in the fabrication process, where the absence of discontinuities ensures the lowest excess loss among those reported in Table 2. As such, we believe our platform sets a new benchmark for optical power splitting in both standard single-mode photonics as well as in the emerging integrated multimode photonics technology. It will enhance important applications – like signal processing, signal feedback and power distribution – that will be substantially boosted by the multiport operation with arbitrary power ratio. Moreover, the ability to split higher-order modes will make these applications available for the multimode photonics and could at the same time enable novel applications for multimode networks.

## Supplementary Material

Supplementary Material Details
