# Supplementary material for: Fabrication of 1 × N integrated power splitters with arbitrary power ratio for single and multimode photonics
Source: Nanophotonics. 2024 Jan 24;13(3):339–48. doi: 10.1515/nanoph-2023-0694 (PMC11501587; doi:10.1515/nanoph-2023-0694)
Supplement: Supplementary file 1 — Supplementary Material Details [file j_nanoph-2023-0694_suppl_001.pdf]

Research Article – Supplementary Material

Jack Haines*, Valerio Vitali, Kyle Bottrill, Pooja Uday Naik, Marco Gandolfi, Costantino De Angelis, Yohann Franz, Cosimo Lacava, Periklis Petropoulos, and Massimiliano Guasoni.

SUPPLEMENTARY: FABRICATION OF 1XN INTEGRATED POWER SPLITTERS WITH ARBITRARY POWER RATIO FOR SINGLE AND MULTI-MODE PHOTONICS

1 Refractive Indices

We report in Fig.S1 the refractive indices of the waveguide material - stoichiometric SiN, (a) - and the Silica cladding (b).

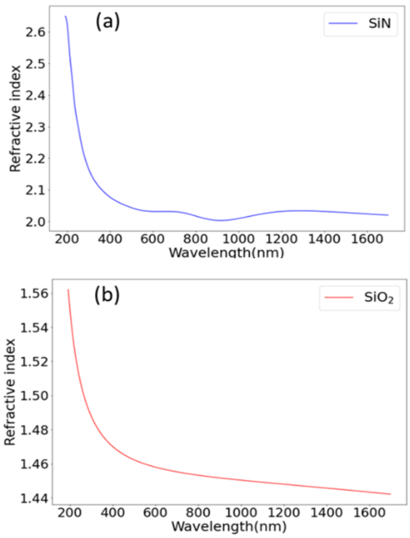

Figure S1: a) Refractive index of LPCVD stoichiometric SiN, measured via a J.A.Woolam M-2000 Ellipsometer provided by Cornerstone Southampton. b) Refractive index of SiO2 used as capping and BOX layer for all devices [1].

2 Additional Power splitters fabricated and tested

Here we discuss the results related to two additional splitters that we have fabricated and tested. Differently from those reported in the main manuscript, these splitters have a thickness of 400 nm. We were unable to reliably characterise the power splitting ratios in the whole bandwidth 1530-1610 nm as well as the excess loss and the modal extinction ratio due to damage at the input facets and the lack of reference straight waveguides or in-series cascades of splitters. Therefore, the results reported here below are preliminary and indicative rather than definitive. The first splitter is a 1x5-TM00 providing an equal splitting ratio. The corresponding splitting ratios and near-field are reported in Fig.S2. An SEM image of this device is displayed in Fig.S3 with a magnified section in panel b that highlights the accurate fabrication of stage-2.
The second device is a 1x5-TE01 designed to provide equal splitting ratio via an integrated mode converter. The corresponding microscope image is reported in Fig.2g-h of the main manuscript. A TE00-like beam is injected at the entry of stage-1, which is converted in a TE01 beam through the directional coupler of stage-1 and then split in the following stages. As it can be seen Fig.S4(d), the near field shows clear presence of the TE01 mode at each output ports. On the other hand, the fields at the outer ports (W_2^{out}, W_3^{out}, W_4^{out}, W_5^{out}) are affected by strong asymmetry in the lobes, which we attribute to the presence of a substantial TE00 component. This issue is currently under investigation.

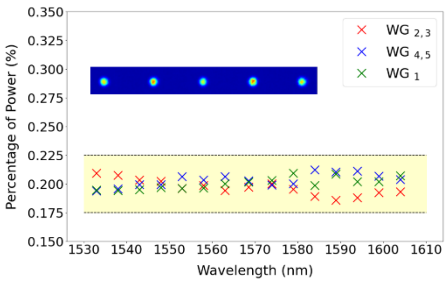

Figure S2: Measured splitting ratios for the 1x5-TM00 power splitter designed to provide equal power splitting (20%) at the output ports. The shaded regions indicate a ±2.5% variation with respect to the nominal splitting ratio of 20%. The inset shows the output near-field at 1532 nm.

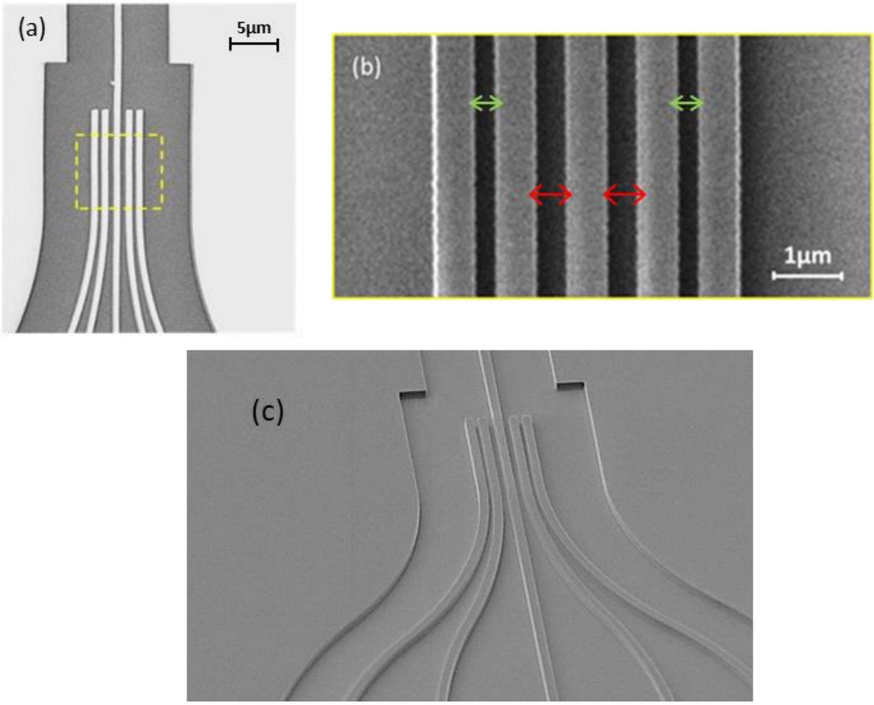

Figure S3: a) SEM image of the 1x5-TM00 power splitter showing all three stages. b) Magnified image of yellow-dashed box in (a), displays a small section of stage 2. Inner gaps (red arrows) are 481 nm wide, outer gaps (green arrows) are 325 nm wide. c) As in panel a but from a different angle. Here, image c, a thin 20nm layer of gold is deposited over the chip to reduce charging and increase resolution.

3 Output Near Fields

Reported in Fig.S4 is an enlarged version of the near-fields displayed in Fig.2(a,c,e,g) of the main manuscript.

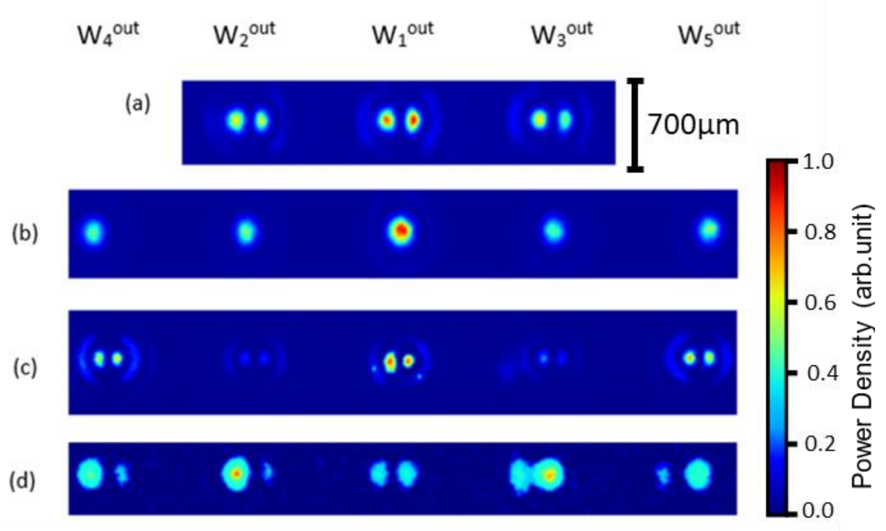

Figure S4: Near-fields at the output ports of the splitters reported in Fig.2 of the main manuscript. From top to bottom (a-d): 1x3-TE01 splitter for equal (1:1:1) splitting; 1x5-TE00 splitter for 1:1:2:1:1 splitting; 1x5-TE01 splitter for 2.5:1:6:1:2.5 splitting; 1x5-TE01 power splitter with integrated mode converter.

4 Testing Power Splitters with Different input Modes

The use of multimode waveguides in our platform brings the opportunity to launch different spatial modes, each one undergoing a different splitting ratio. In other words, it is possible to obtain different splitting ratios within the same device. As an example, let us consider the 1x3-TE01 power splitter discussed in Fig.6a of the main manuscript. In Table S1 we report the different power splitting ratios obtained when coupling a TE00, TM00 and TE01 beam at the entry of the splitter (input of stage 1). Note that because the TE00 mode is tightly confined into the waveguides, the coupling dynamics is strongly suppressed and almost all the power remains confined in the central waveguide W1.

Also note that, as anticipated in the main manuscript, the difference between the powers measured at output ports W_2^{out} and W_3^{out} is as small as < 1% (the same applies to the pair of ports W_4^{out} and W_5^{out} in 1x5 splitters).

	TE00		TE01		TM00	
	EXP (%)	SIM (%)	EXP (%)	SIM (%)	EXP (%)	SIM (%)
Port $W1_{out}$	92.5	94.6	34	31	79	78
Port $W2_{out}$	3.9	2.7	33.3	34.5	10.6	11
Port $W3_{out}$	3.6	2.7	32.7	34.5	10.4	11

Table S1: Power ratios (in %) at the output ports of the 1x3 splitter discussed in Fig.6a of the main manuscript for different input modes and at wavelength of 1550 nm. Comparison among experimental measurements (Exp) and simulation results (SIM).

References

1] I. Malitson, "Interspecimen Comparison of the Refractive Index of Fused Silica," J. Opt. Soc. Am. 55, 1205-1209 (1965).
